# Maternal Plasma Fetal DNA Fractions in Pregnancies with Low and High Risks for Fetal Chromosomal Aneuploidies

**DOI:** 10.1371/journal.pone.0088484

**Published:** 2014-02-28

**Authors:** Irena Hudecova, Daljit Sahota, Macy M. S. Heung, Yongjie Jin, Wing S. Lee, Tak Y. Leung, Yuk Ming Dennis Lo, Rossa W.K. Chiu

**Affiliations:** 1 Centre for Research into Circulating Fetal Nucleic Acids, Li Ka Shing Institute of Health Sciences, The Chinese University of Hong Kong, Shatin, New Territories, Hong Kong SAR, China; 2 Department of Chemical Pathology, The Chinese University of Hong Kong, Prince of Wales Hospital, Shatin, New Territories, Hong Kong SAR, China; 3 Department of Obstetrics and Gynaecology, The Chinese University of Hong Kong, Prince of Wales Hospital, Shatin, New Territories, Hong Kong SAR, China; Inra, France

## Abstract

Recently published international guidelines recommend the clinical use of noninvasive prenatal test (NIPT) for aneuploidy screening only among pregnant women whose fetuses are deemed at high risk. The applicability of NIPT to aneuploidy screening among average risk pregnancies requires additional supportive evidence. A key determinant of the reliability of aneuploidy NIPT is the fetal DNA fraction in maternal plasma. In this report, we investigated if differences in fetal DNA fractions existed between different pregnancy risk groups. One hundred and ninety-five singleton pregnancies with male fetuses divided into 3 groups according to first trimester screening parameters were examined for fetal DNA percentage by counting Y chromosome DNA sequences using massively parallel sequencing. Fetal DNA fractions were compared between risk groups and assessed for correlations with first trimester screening parameters. There was no statistically significant difference in fetal DNA fractions across the high, intermediate and low risk groups. Fetal DNA fraction showed a strong negative correlation with maternal weight. Fetal DNA fraction also showed weak but significant correlations with gestational age, crown-rump length, multiple of medians of free β-subunit of human chorionic gonadotropin and pregnancy-associated plasma protein A. Similar fetal DNA fractions in maternal plasma between high, intermediate and low risk pregnant women is a precondition for uniform performance of the aneuploidy NIPTs for the general population. This study thus shows that the aneuploidy screening by NIPT is likely to offer similar analytical reliability without respect to the *a priori* fetal aneuploidy risk.

## Introduction

Noninvasive fetal chromosomal aneuploidy screening based on massively parallel sequencing has been validated in multiple clinical trials and has been shown to be highly sensitive and specific for patients at increased risk of fetal aneuploidies [1]–[6]. Recently, international guidelines on aneuploidy screening by noninvasive prenatal test (NIPT) published by the American College of Obstetricians and Gynecologists (ACOG), the American College of Medical Genetics and Genomics (ACMG) and the International Society for Prenatal Diagnosis support the use of the approach for high risk pregnancies [7]–[9]. Pregnancies deemed at high risk include women aged 35 or above, with ultrasonographic findings indicating an increased risk of fetal aneuploidy, previous pregnancy affected with aneuploidy or parental balanced robertsonian translocation associated with trisomy 21 and 13, as well as those screened positive by conventional first or second trimester screening tests. However, there is less certainty regarding the applicability of the NIPT screening test to the general population, i.e. women at average risk.

Determination of fetal trisomies using NIPT relies on the detection of a small increment in sequence reads aligned to a specific chromosome, statistically expressed by the z score. Sufficient sequencing data have to be obtained to reveal such a deviation from the euploid reference data set. Fetal DNA fraction is a key parameter in determining the robustness for fetal chromosomal status assessment. The fetal DNA fraction governs the degree of deviation in the proportion of sequence reads from the aneuploid chromosome if fetal aneuploidy is present. Various groups have attempted to verify the test performance in the general pregnant population [10]–[12]. Most of these studies explored the diagnostic performance in the general population without detailed assessment of the fetal DNA fraction in maternal plasma or did not make direct comparisons with high risk cases. A recent study focused on a subpopulation of high risk pregnancies [13] by selecting cases with values at the two ends of the spectrum for each individual marker that defined the risk. The authors then examined the fetal DNA fractions among those subpopulations covering a broad gestational age range (10 to 38 weeks of gestation).

Ashoor et al studied parameters that influenced the fetal DNA fraction among average risk women in the first trimester [14]. The data did not appear to differ from those obtained from high risk women. The goal of the present study is to systematically compare fetal DNA fractions between different risk groups by counting Y chromosome sequences on a cohort of pregnancies with male fetuses that were recruited from a routine first trimester screening clinic. In addition, we examine if a relationship between fetal DNA fraction and the first trimester screening (FTS) parameters existed.

## Materials and Methods

### Ethics statement

Ethical approval was obtained from the Joint New Territories East Cluster–Chinese University of Hong Kong Clinical Research Ethical Committee and all participants gave informed written consent.

### Sample collection and recruitment criteria

We consecutively recruited consenting pregnant women from the FTS clinic at the Department of Obstetrics and Gynaecology, The Chinese University of Hong Kong, Hong Kong [15], between November 2011 and January 2013. The inclusion criteria were male singleton pregnancies with recorded data on FTS parameters for risk group stratification. We defined the high risk group (HR) as those pregnancies whose determined risk for trisomy 21 is greater than 1 in 250, the intermediate risk group (IR) were pregnancies with risks between 1 in 250 to 1 in 1000 and the low risk group (LR) were pregnancies with risks lower than 1 in 1000.

### Plasma processing

Blood samples were collected in EDTA-containing tubes before invasive obstetric procedures and processed by double-centrifugation before storage at −80°C. We extracted plasma DNA from 4 mL of stored plasma sample using the protocol reported previously [1].

### Plasma DNA sequencing and data analysis

DNA sequencing libraries were prepared using the TruSeq DNA Sample Prep Kit (Illumina, Inc). Unique DNA barcode sequences, referred as indices, were introduced to allow pooled sequencing runs. We used 12 different indices to distinguish twelve maternal plasma samples in a 12-plex sequencing protocol. A total of 18 lanes on flow cells were sequenced on a HiSeq 2000 sequencer (Illumina) using standard single-end 36 bp reads. Sequencing reads were mapped to the nonrepeat-masked reference human genome (hg18) using the SOAP2 bioinformatic algorithm as previously described [1]. We included only reads without mismatches and reads deemed as unique in the reference human genome. The number of unique sequence reads aligned to each chromosome was counted. These values were used to calculate the percentage contribution (also referred as genomic representation, GR) of unique reads for each chromosome separately.

As reported previously [1], we determined the fetal DNA fraction in maternal plasma by taking into account the amount of chromosome Y sequences contributed by the male fetus and sequences originated from the maternal background DNA that were incorrectly assigned to chromosome Y. Thus, we measured the mean fraction of chromosome Y sequence reads of plasma samples obtained from adult male individuals (*male %chrY*) and from pregnant women bearing euploid female fetuses (*female %chrY*) to calculate the fetal DNA fraction (*F*):
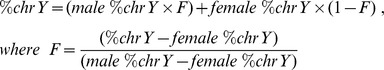



For each case which passed the recruitment and quality control criteria (samples with sequence read counts between the 1^st^ and 99^th^ centile of the whole cohort) [1], we calculated the z score expressed as a number of standard deviations in chromosome percentage for the test sample from the mean of an euploid reference data set. To assess the fetal chromosomal status, a cut-off >3 has been chosen. We then determined sensitivity and specificity of the test by comparing the sequencing results and outcomes obtained after the birth or from karyotyping of the amniotic fluid or chorionic villus sampling. We calculated the coefficient of variation (CV) for each chromosome in the reference data set as a ratio of standard deviation (SD) and mean genomic representation.

### Statistical analysis

Fetal DNA fractions were compared between the three fetal aneuploidy risk groups (HR, IR, LR) and assessed for correlation with the FTS test parameters; gestational age, crown-rump length (CRL), free β-subunit of human chorionic gonadotropin (free β-hCG), nuchal translucency (NT), pregnancy-associated plasma protein A (PAPP-A) as well as maternal weight using SigmaStat (version 3.5) software and visualized by SigmaPlot 8.0 and MedCalc (version 11.4.4.0). The measured free β-hCG, NT and PAPP-A were converted into multiples of the median (MoM) and log_10_ transformed to obtain a Gaussian distribution. Normality was assessed by the Kolmogorov-Smirnov Z test. We examined differences among groups using analysis of variance test (ANOVA) or Mann-Whitney Rank Sum Test as appropriate, with P value <0.05 to indicate statistical significance. Regression analysis (Pearson's Product-Moment Correlation) was used to assess the relationship between fetal DNA fraction and maternal weight as well as FTS parameters (gestational age, CRL, log_10_ free β-hCG MoM, log_10_ NT MoM, log_10_ PAPP-A MoM).

## Results

### Maternal plasma fetal DNA fractions in different risk groups

Among the 726 pregnancies recruited, we selected for singleton pregnancies with male fetuses confirmed by ultrasonography and maternal plasma *sex chromosome region Y* (*SRY*) assessment by real-time polymerase chain reaction [16]. In total 337 cases met this requirement. We then stratified samples into 3 groups according to the calculated risk of fetal trisomy determined by the FTS test. The minimum number of samples required to detect lack of differences in fetal DNA fraction with a 99% statistical power at 0.05 type I error was found to be 127. We therefore selected 138 LR pregnancies with male fetuses. Because the recruitment took place among the general population, the number of IR or HR pregnancies was few. We therefore included all the IR and HR pregnancies with male fetuses that were identified in the cohort (Table 1). We also included all the 7 trisomy 21 (T21) cases (2 females and 5 males) and 1 trisomy 18 (male) detected in the cohort. We collected plasma samples from two adult male volunteers to determine the fraction of chromosome Y sequence reads (%chrY) corresponding to plasma samples with 100% male DNA. Ten pregnancies involving female fetuses were analyzed to provide the baseline amount of chromosome Y sequences that could be detected in 100% female plasma DNA due to sequence misalignment [1]. Table 1 summarizes the number of cases included in this study. Table 2 summarizes the demographics and first trimester screening results of the studied cohort. The mean maternal age was 32 years and the mean gestational age was 12 weeks and 5 days.

**Table 1:** Overview of samples.

Case	Risk group		Number of samples
Adult male	Control		2
Female fetus	Control		10
Male fetus	High risk (>1∶250)		22
	Intermediate risk (1∶1000–1∶250)		36
	Low risk (<1∶1000)		138
T21 fetus	High risk	Male fetus	5
		Female fetus	2
T18 fetus	Intermediate risk		1
TOTAL			216

**Table 2:** First trimester screening test parameters for the high, intermediate and low risk pregnancies for cases meeting quality control criteria for chromosome Y assessment (N = 195).

First trimester screening	Risk group	Mean	Std Dev	Median	Range	IQR^a^
Trisomy 21 risk	HR	1∶74	-	1∶49	1∶2–1∶243	1∶4–1∶136
	IR	1∶619	-	1∶678	1∶259–1∶983	1∶375–1∶871
	LR	1∶10 881	-	1∶8 786	1∶1 026–1∶28 832	1∶4 057–1∶16 944
Maternal age (years)	HR	35.3	4.1	36.0	27.0–43.0	33.0–38.0
	IR	34.4	4.3	35.0	23.0–44.0	32.0–37.0
	LR	31.1	4.0	31.0	17.0–41.0	29.0–34.0
USG Scan Gest^b^ (days)	HR	88.0	4.0	89.0	81.0–97.0	85.0–91.0
	IR	90.0	4.0	90.0	80.0–97.0	86.0–93.0
	LR	89.0	3.0	89.0	80.0–97.0	87.0–91.0
Maternal weight (kg)	HR	57.5	8.7	55.3	45.0–74.0	51.8–64.1
	IR	56.3	7.9	57.1	41.5–74.5	49.5–60.5
	LR	56.3	8.2	55.5	41.5–86.2	50.5–60.4
CRL^c^ (mm)	HR	62.4	8.8	62.9	48.9–82.8	55.9–68.6
	IR	65.2	8.4	65.9	46.3–80.2	60.2–71.2
	LR	62.9	6.9	63.2	46.7–81.1	58.7–68.0
Free β-hCG MoM^d^	HR	2.37	1.23	1.96	0.63–5.56	1.51–2.86
	IR	2.11	1.53	1.64	0.37–8.10	1.04–2.86
	LR	1.11	0.65	0.94	0.22–4.49	0.70–1.34
PAPP-A MoM^e^	HR	0.74	0.30	0.71	0.25–1.35	0.49–0.92
	IR	0.82	0.43	0.72	0.14–1.97	0.49–1.13
	LR	1.09	0.58	0.97	0.31–3.50	0.67–1.33
NT MoM^f^	HR	1.88	0.97	1.63	0.97–5.43	1.31–1.87
	IR	1.14	0.28	1.07	0.67–1.81	0.97–1.29
	LR	1.02	0.19	1.02	0.55–1.54	0.89–1.16

HR, high risk; IR, intermediate risk; LR, low risk.

a IQR, interquartile range.

b USG Scan Gest, gestational age based on ultrasonography scanning.

c CRL, crown-rump length.

d Free β-hCG MoM, multiples of the median of free β-subunit of human chorionic gonadotropin.

e PAPP-A MoM, multiples of the median of pregnancy-associated plasma protein A.

f NT MoM, multiples of the median of nuchal translucency.

Among the selected pregnancies, four cases did not meet the recruitment criteria because they were later confirmed to be female fetuses but had incorrect fetal sex entries in the database. Three samples (1.5%) did not meet quality control requirements after sequencing. Thus, in total 195 male fetuses (135 LR, 35 IR, 25 HR) had been included in the assessment of the fetal DNA fractions.

**Figure 1:**
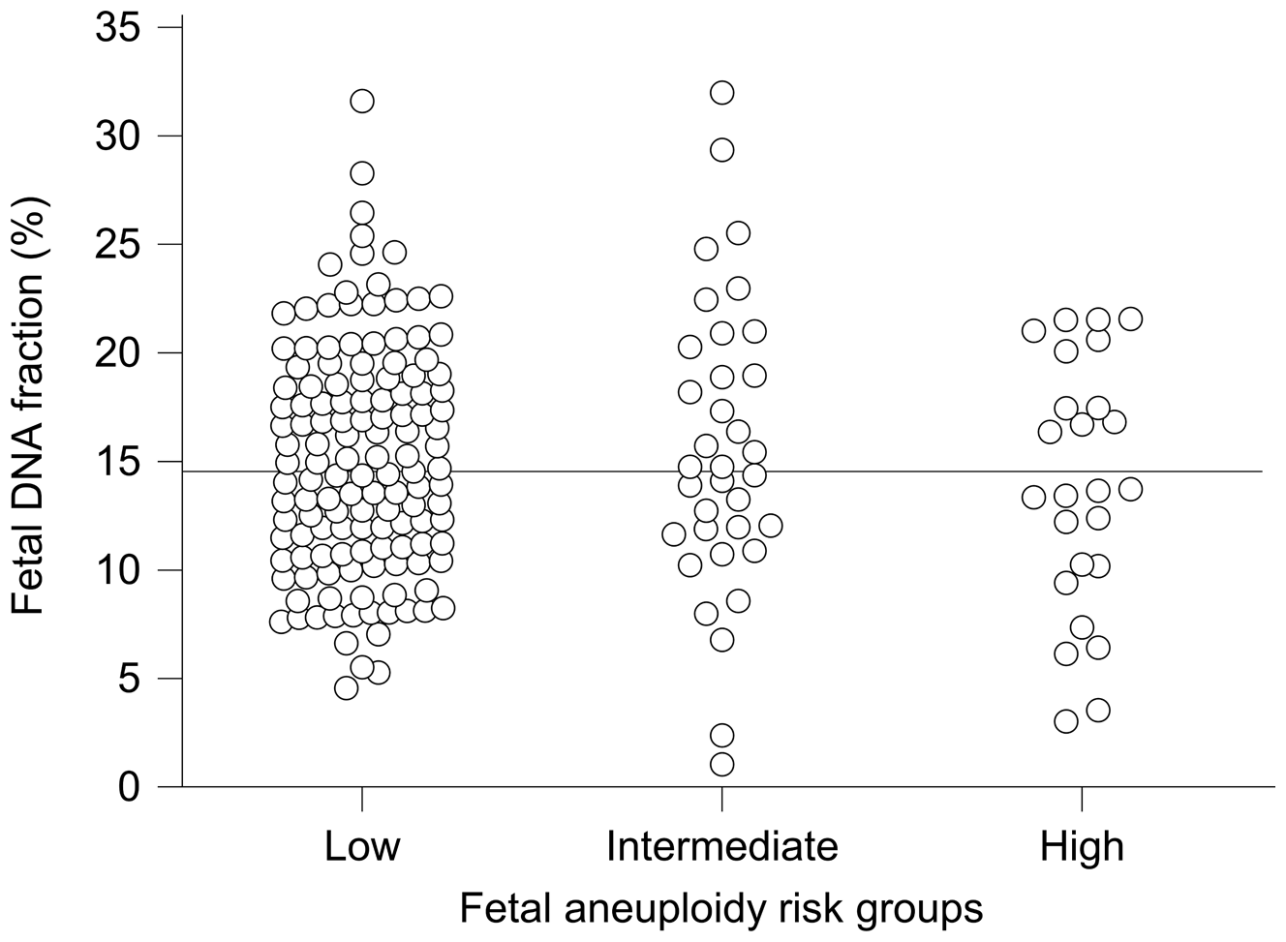
Fetal DNA fractions among pregnancy risk groups. Horizontal line shows the overall median fetal DNA fraction.

The fetal DNA fractions for all cases among the three risk groups are depicted graphically in Figure 1. The median fetal DNA fraction was 14.5% (interquartile range 10.9%–18.9%) for the whole cohort, 13.6% (interquartile range 10.0%–18.1%) for the HR group, 14.8% (interquartile range 11.7%–20.0%) for the IR group and 14.7% (interquartile range 11.0%–18.7%) for the LR group. Among the LR, IR and HR pregnancies recruited in this study, there were no statistically significant differences among the fetal DNA fractions (ANOVA, P = 0.490).

**Figure 2:**
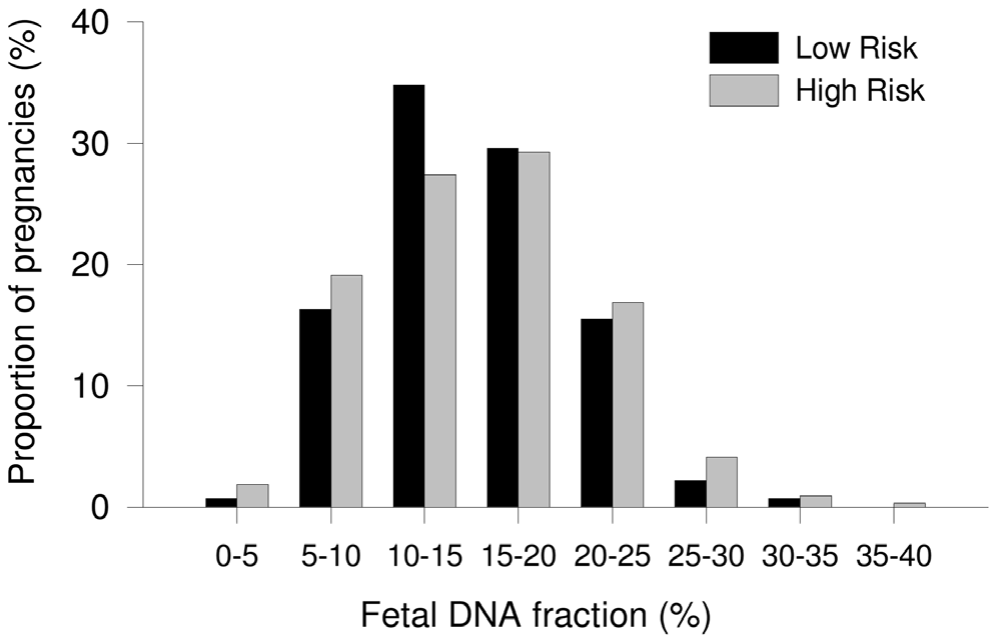
Frequency distributions of the range of fetal DNA fractions found in male euploid pregnancies. Fetal DNA fractions are compared among pregnancies at high or low risk for trisomy 21.

We next investigated the frequency distribution of the range of fetal DNA fractions found (range 0%–40% divided into intervals of 5%). We compared the data from 135 LR pregnancies with those published previously [1] on the cohort of 314 HR pregnancies (median 16.7%, interquartile range 11.3%–21.5%). The median gestational ages for LR and HR cohort were 12.4 weeks (interquartile range 12.3–12.7) and 13.1 weeks (12.5–14.0), respectively. Figure 2 shows that there was no statistically significant difference in the distributions of fetal DNA fractions among male euploid pregnancies in the HR and LR groups (t-test, P = 1.0). The fetal DNA fractions between our LR group and HR group from the previous study did not reach statistical significance as well (Mann-Whitney, P = 0.736).

### Comparison of fetal DNA fractions in maternal plasma with first trimester screening parameters

**Figure 3:**
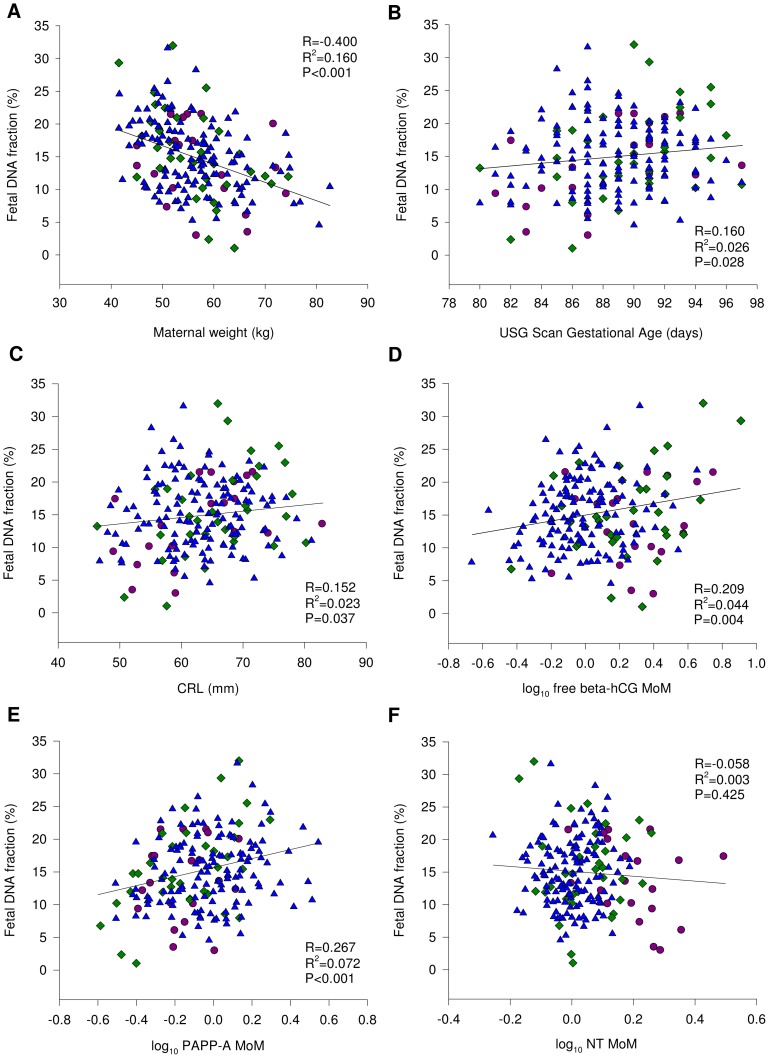
Relationships between fetal DNA fractions with the first trimester screening parameters between pregnancy risk groups. Depicted are pregnancies at high risk (purple circles), intermediate risk (green diamonds) and low risk (blue triangles).

We studied if there was any correlation between the fetal DNA fractions and maternal weight as well as each of the FTS parameters, namely gestational age, CRL, log_10_ free β-hCG MoM, log_10_ NT MoM, and log_10_ PAPP-A MoM. The data are shown in Figure 3. We found statistically significant negative correlation between fetal DNA fraction and maternal weight, the Pearson correlation R^2^ = 0.160, (P<0.001) (Figure 3A). There seemed to be statistically significant relationships between the fetal DNA fraction and the gestational age (Figure 3B), the CRL (Figure 3C), log_10_ free β-hCG MoM (Figure 3D) and log_10_ PAPP-A MoM (Figure 3E). However, the correlations were weak. For gestational age, the Pearson correlation R^2^ was 0.026 (P = 0.028), CRL, the Pearson correlation R^2^ was 0.023 (P = 0.037), log_10_ free β-hCG MoM, the Pearson correlation R^2^ was 0.044, (P = 0.004) and log_10_ PAPP-A MoM, the Pearson correlation R^2^ was 0.072 (P<0.001). We did not detect any statistically significant relationship between fetal DNA fraction and log_10_ NT MoM (Figure 3F) (Pearson correlation R^2^ = 0.003, P = 0.425).

### Noninvasive fetal trisomy 21 assessment

Of the 214 plasma samples included in our cohort, 7 cases did not meet the study criteria; namely 4 samples (1.9%) which did not fulfill recruitment criteria and 3 samples (1.4%) with sequencing read counts beyond the central 99^th^ centile for the cohort. Prenatal karyotyping revealed presence of 7 T21 cases (2 females, 5 males), one pregnancy with a male T18 fetus and 199 euploid pregnancies confirmed after birth. All T21 cases were among the high risk group, one T18 case was classified as at intermediate risk. For the z score calculation, we randomly selected 30 pregnancies with euploid male fetuses as a reference sample set. The remaining 177 samples (12 females, 165 males) had been tested for the fetal chromosomal status.

On average, 3.7 M (SD 1.3 M) sequence reads per sample were obtained. The coefficient of variation for chromosome 21 using a 12-plex sequencing protocol calculated from the control set (30 euploid male fetuses) was 0.56% (SD 0.31%). We found 2 IR euploid cases and 2 HR euploid cases to be below the fetal DNA fraction cutoff of 4% [3]. Of the remaining cases, we detected all 7 T21 cases with a false-positive rate of 1.8% (3/166).

**Figure 4:**
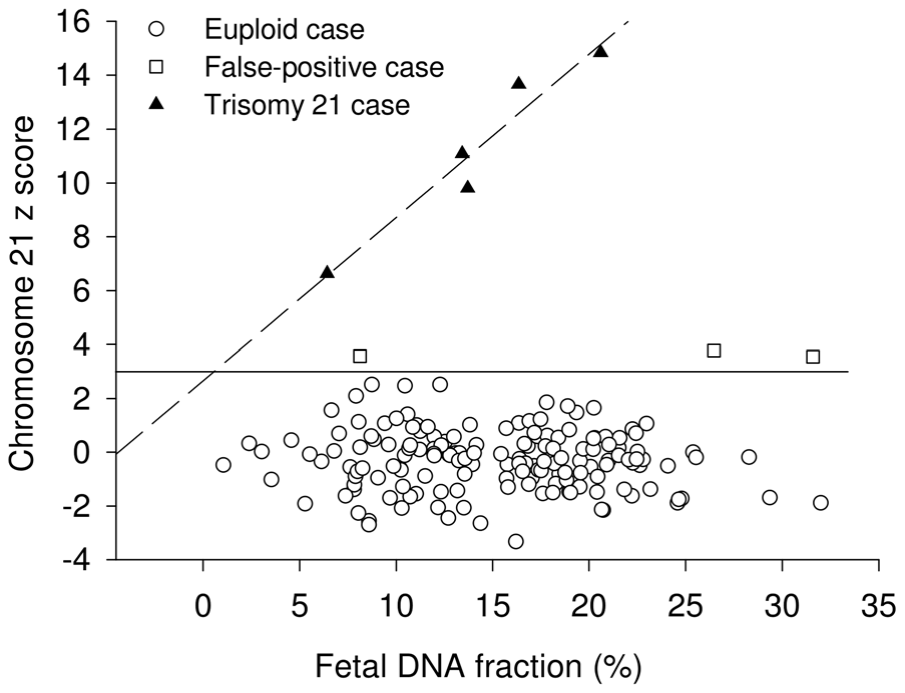
Relationship of the z score with fetal DNA fractions in maternal plasma. Solid line marks the z score cutoff of 3 for detecting elevated proportions of chromosome 21. Dash line shows the correlation between the z score and fetal DNA fractions for the trisomy 21 cases.

We plotted the z scores for all tested cases, excluding the 30 cases included in the reference sample set, against the fetal DNA percentage calculated from %chrY (Figure 4). We expect a positive correlation between the z score of the aneuploid chromosome and fetal DNA fractions in maternal plasma in cases showing true positive sequencing results for fetal aneuploidy [1], [3]. Indeed, for all T21 cases a significant correlation has been observed, R^2^ was 0.94 (P = 0.007). The three false-positive cases did not fall on the correlation line (Figure 4). The protocol for fetal trisomy 18 detection confirmed trisomy 18 in one of the fetuses (z score chr18 = 4.2) with no false positive cases.

## Discussion

Here, we report the data on fetal DNA fractions between 3 risk groups of pregnancies stratified according to the FTS results. We compared the fetal DNA fractions between high, intermediate and low risk groups and did not find any statistically significant differences. In addition, we compared the fetal DNA fractions of the 135 LR pregnancies of this study with the 314 HR pregnancies assessed in our previous study [1]. In both, the fetal DNA fractions were measured using the same calculations based on the amounts of chromosome Y reads detected by maternal plasma DNA sequencing. We believe that the large LR and HR case numbers studied in each of the two studies; and the precise and standardized means used to measure the fetal DNA fractions, provided strong evidence for the lack of difference in fetal DNA fractions between these groups. The impact of fetal DNA proportion in maternal plasma has been established as a critical determinant of the NIPT aneuploidy test performance [17]. Thus, these data suggest that aneuploidy screening by NIPT is likely to offer similar analytical performance across the high, intermediate and low risk pregnancies.

We further investigated if any strong correlation existed between fetal DNA fraction and maternal weight and each FTS parameter; namely gestational age, CRL, free β-hCG MoM, NT MoM and PAPP-A MoM. A strong negative correlation between fetal DNA fraction and maternal weight was observed as reported in earlier studies [14], [17], [18]. It is interesting that this relationship was observed even in this cohort of ethnically Chinese women with a relatively narrow weight range (range: 41.5 kg to 86.2 kg (Table 2)). The data indicate the importance of establishing a cutoff for fetal DNA fraction to identify cases with inadequate fetal DNA for noninvasive fetal aneuploidy assessment, particularly among the heavier women.

While the relationship with fetal DNA fraction was statistically significant for gestational age, CRL, log_10_ free β-hCG MoM and log_10_ PAPP-A MoM, the degree of correlation was weak (Figure 3). Amongst these relatively weak correlations, the relationship between fetal DNA fraction and log_10_ PAPP-A MoM was comparatively prominent. The trend appeared to be a positive correlation and may be a result of both PAPP-A and cell-free fetal DNA being derived from the placenta.

Compared with the previous studies [6], [13], one strength of our study is the prospective recruitment of women presenting for first trimester fetal aneuploidy screening and our specific focus on low risk pregnancies. In addition, the gestational age range of the included patients corresponded to the gestational age window when the actual test is required (mean 12 weeks and 5 days). We used massively parallel sequencing to obtain precise measurements of the maternal plasma fetal DNA fractions for all studied cases. A large-scale multicenter study performed by Dan et al on 11 105 pregnancies included a subpopulation of cases from non-HR pregnancies [11]. However, the distribution of fetal DNA fractions among these pregnancies was not studied in detail. A comprehensive study on the first-trimester general population was conducted by Nicolaides et al [12] but there were limited data on fetal DNA fraction among each risk group. The relationship of fetal DNA fraction in maternal plasma with FTS parameters has been studied by Ashoor et al who initially focused on HR pregnancies [18]. Their subsequent study focused on a large cohort of pregnant women attending the first trimester screening clinic. Our findings on the relationships between fetal DNA fractions and FTS parameters were similar to those of Ashoor et al [14]. However, a detailed comparison of fetal DNA fractions between risk groups was not reported in the earlier study [14].

In summary, the present study provides evidence based on measurements of maternal plasma fetal DNA fraction that the NIPT aneuploidy test is likely to perform analytically as well in different risk groups of pregnant women. Despite the same analytical performance of the test across risk groups, the positive predictive value of the test would be reduced due to the lower prevalence of chromosome aneuploidy in the average risk population compared to the high risk group. Therefore, whether NIPT for chromosome aneuploidy detection should be applied to average risk women is mainly a cost-benefit question rather than an issue of its analytical performance. To formally address the diagnostic performance and cost-benefits of the NIPT aneuploidy screening test among the general population, a large scale prospective study could be considered. Due to the low prevalence of chromosomal aneuploidy in the general population, extremely large sample size would be needed.
